# Supplementary figures and images for: Comparative genomic analysis of the IDD genes in five Rosaceae species and expression analysis in Chinese white pear (Pyrus bretschneideri)
Source: PeerJ. 2019 Mar 26;7:e6628. doi: 10.7717/peerj.6628 (PMC6440465; doi:10.7717/peerj.6628)

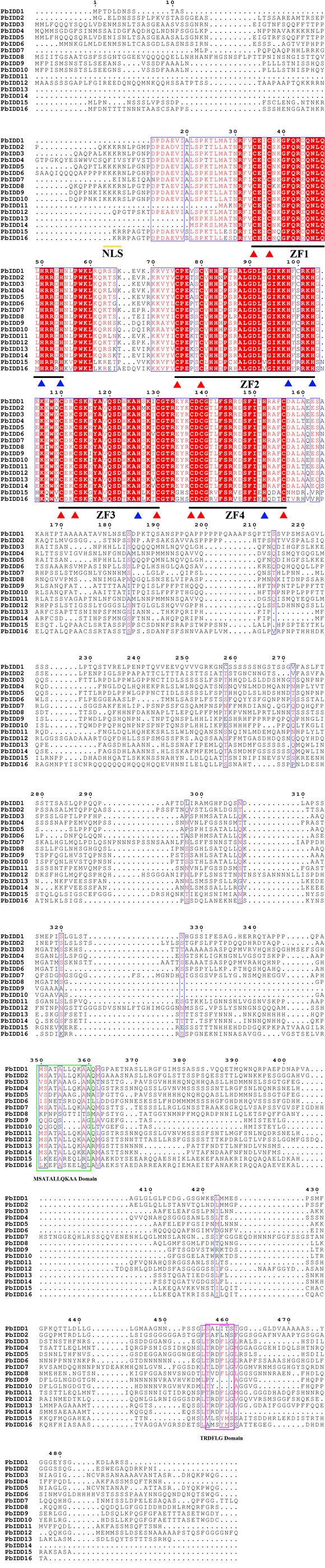

Supplement: Supplemental Information 1 — Black underline indicates zinc finger domain (Z1, Z2, Z3 and Z4). Red triangle indicates a conserved C residue, and blue triangle indicates a conserved H residue. The yellow underline indicates the NLS sequence in the N-terminal region of the IDD gene. Green box means the MSATALLQKAA domain, and purple box indicates the TRDFLG domain. [file peerj-07-6628-s001.png]

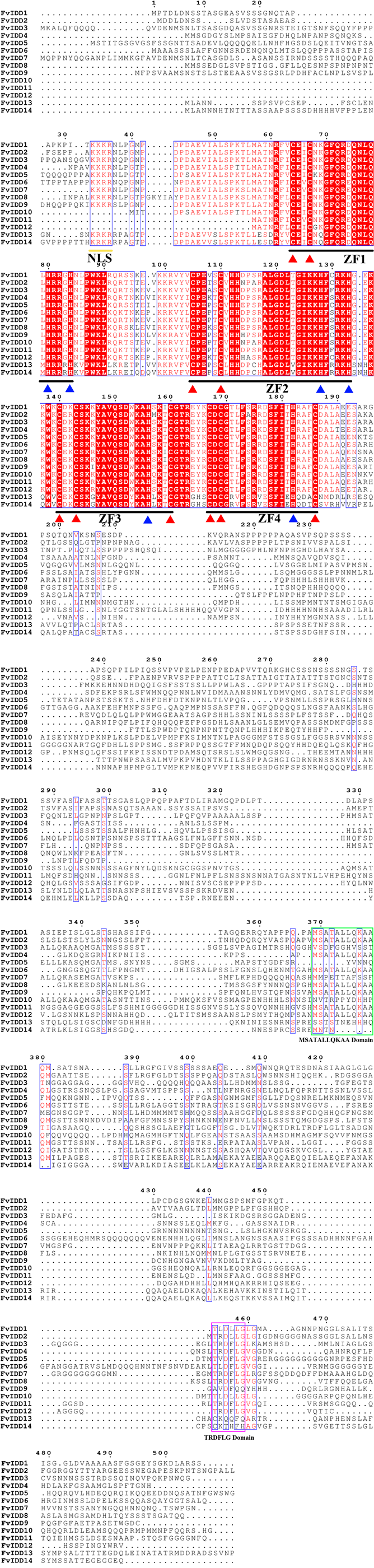

Supplement: Supplemental Information 2 — Black underline indicates zinc finger domain (Z1, Z2, Z3 and Z4). Red triangle indicates a conserved C residue, and blue triangle indicates a conserved H residue. The yellow underline indicates the NLS sequence in the N-terminal region of the IDD gene. Green box means the MSATALLQKAA domain, and purple box indicates the TRDFLG domain. [file peerj-07-6628-s002.png]

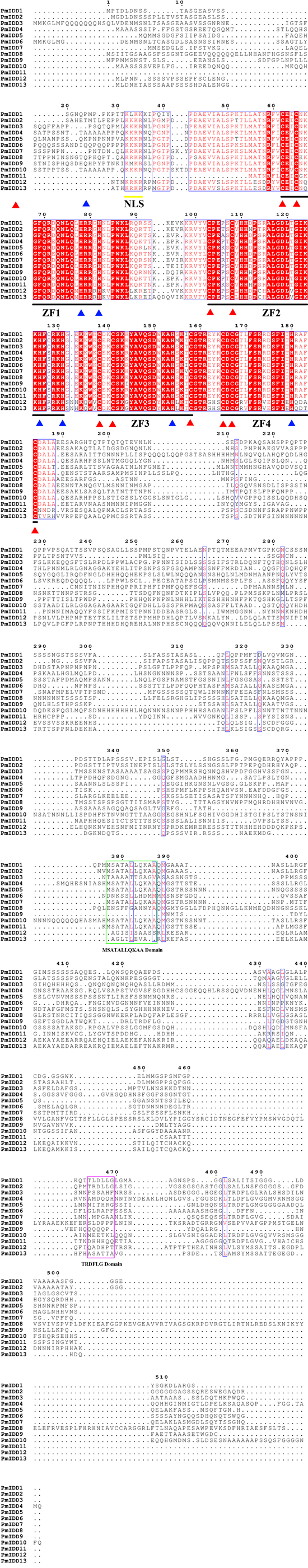

Supplement: Supplemental Information 3 — Black underline indicates zinc finger domain (Z1, Z2, Z3 and Z4). Red triangle indicates a conserved C residue, and blue triangle indicates a conserved H residue. The yellow underline indicates the NLS sequence in the N-terminal region of the IDD gene. Green box means the MSATALLQKAA domain, and purple box indicates the TRDFLG domain. [file peerj-07-6628-s003.png]

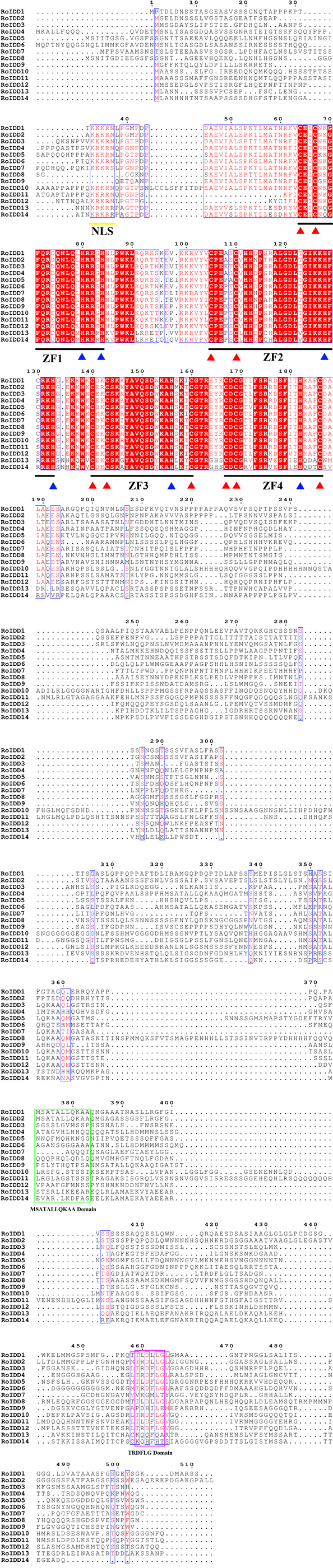

Supplement: Supplemental Information 4 — Black underline indicates zinc finger domain (Z1, Z2, Z3 and Z4). Red triangle indicates a conserved C residue, and blue triangle indicates a conserved H residue. The yellow underline indicates the NLS sequence in the N-terminal region of the IDD gene. Green box means the MSATALLQKAA domain, and purple box indicates the TRDFLG domain. [file peerj-07-6628-s004.png]

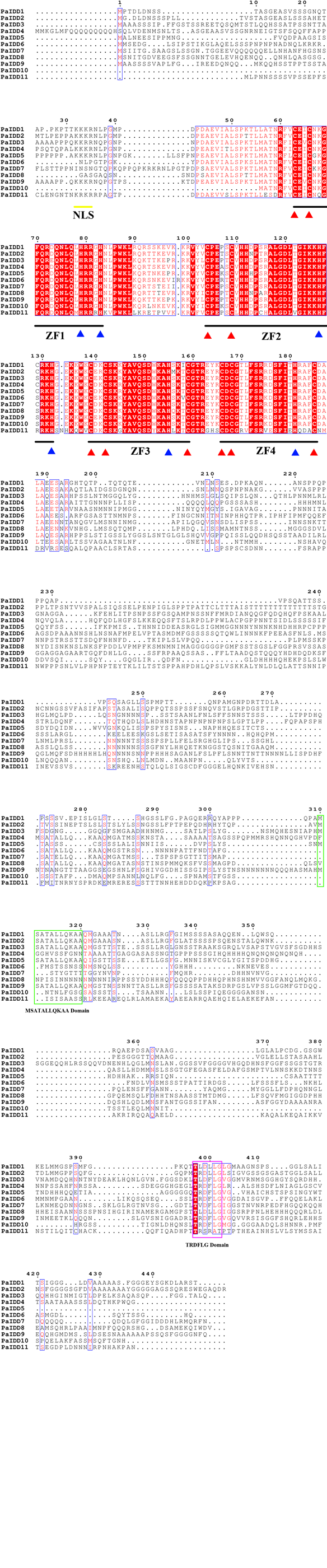

Supplement: Supplemental Information 5 — Black underline indicates zinc finger domain (Z1, Z2, Z3 and Z4). Red triangle indicates a conserved C residue, and blue triangle indicates a conserved H residue. The yellow underline indicates the NLS sequence in the N-terminal region of the IDD gene. Green box means the MSATALLQKAA domain, and purple box indicates the TRDFLG domain. [file peerj-07-6628-s005.png]

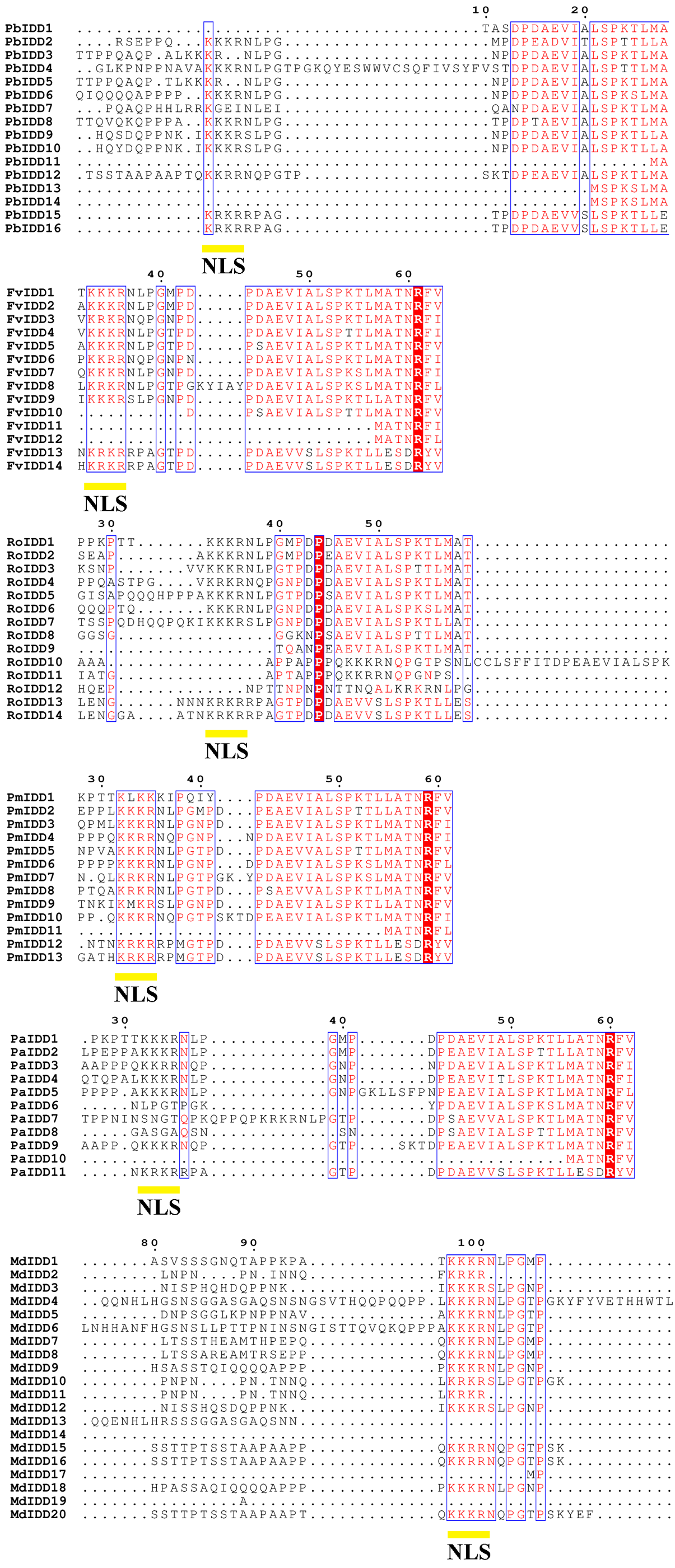

Supplement: Supplemental Information 6 — The yellow underline indicates the NLS sequence in the N-terminal region of the IDD gene. [file peerj-07-6628-s006.png]

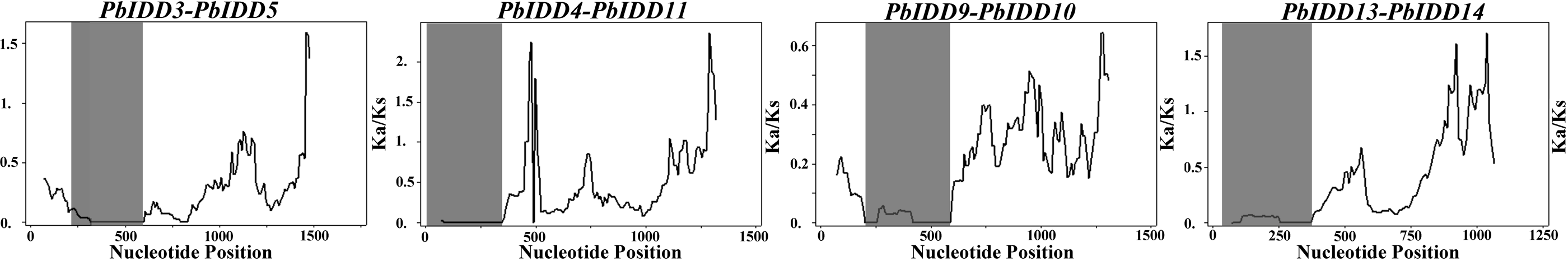

Supplement: Supplemental Information 7 — The grey shaded portion indicates conserved ID domain. The X-axis indicates the synonymous distance within each gene. [file peerj-07-6628-s007.png]

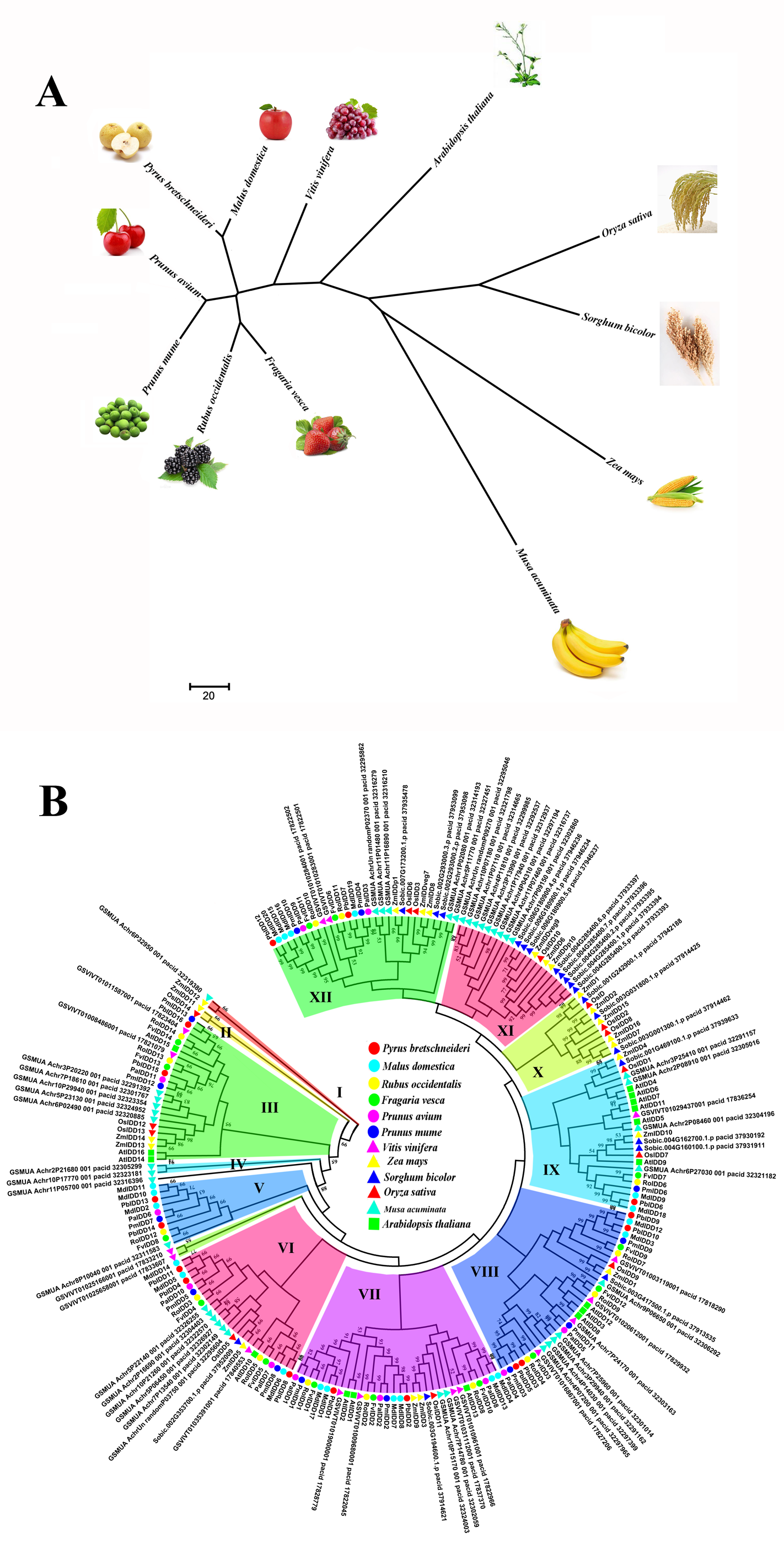

Supplement: Supplemental Information 8 — A phylogenetic tree of the 12 species genomes (A). Phylogenetic relationships and subfamily designations in IDD proteins from 12 species (B). [file peerj-07-6628-s008.png]

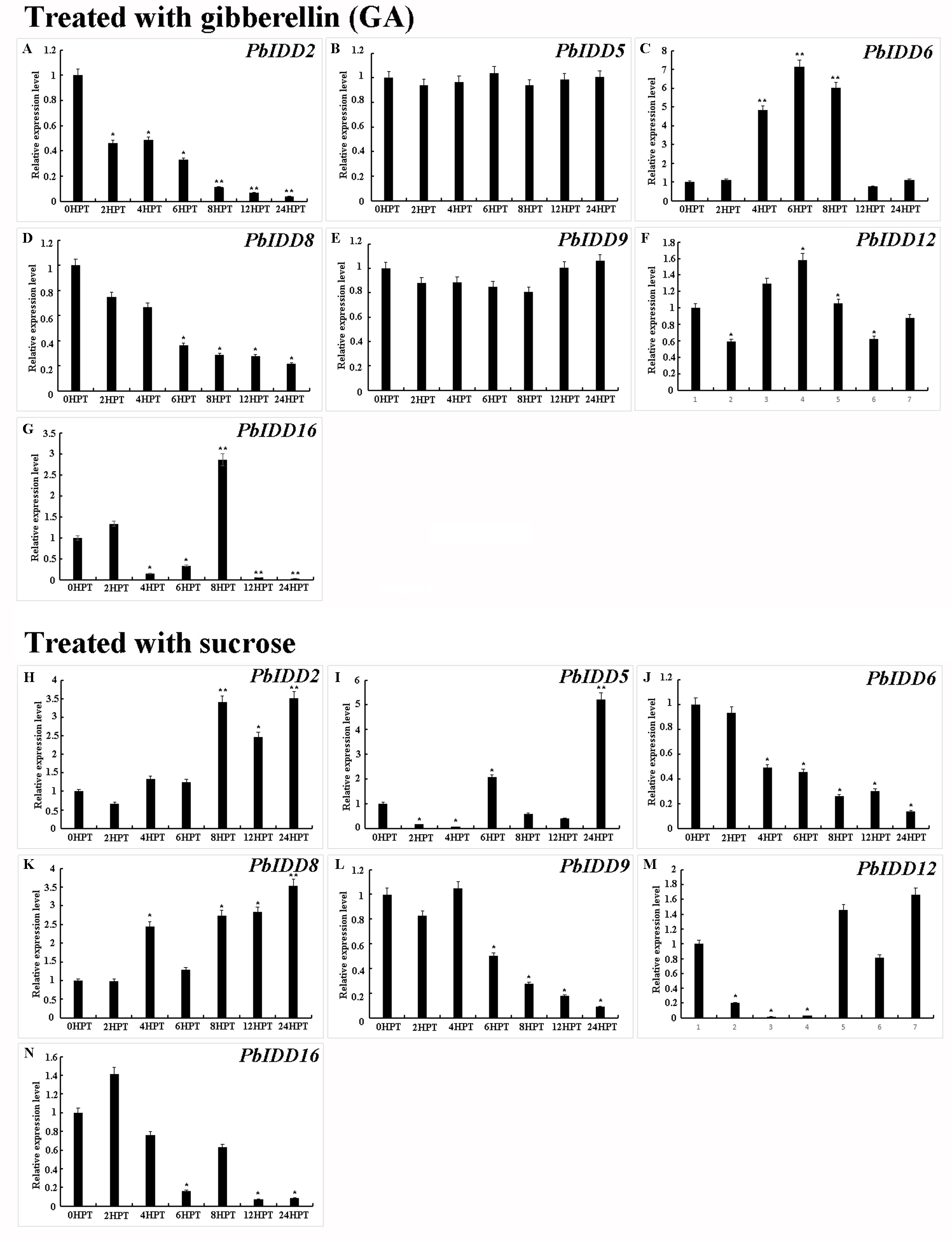

Supplement: Supplemental Information 9 — *significant difference at P < 0.05, **significant difference at P < 0.01. [file peerj-07-6628-s009.png]

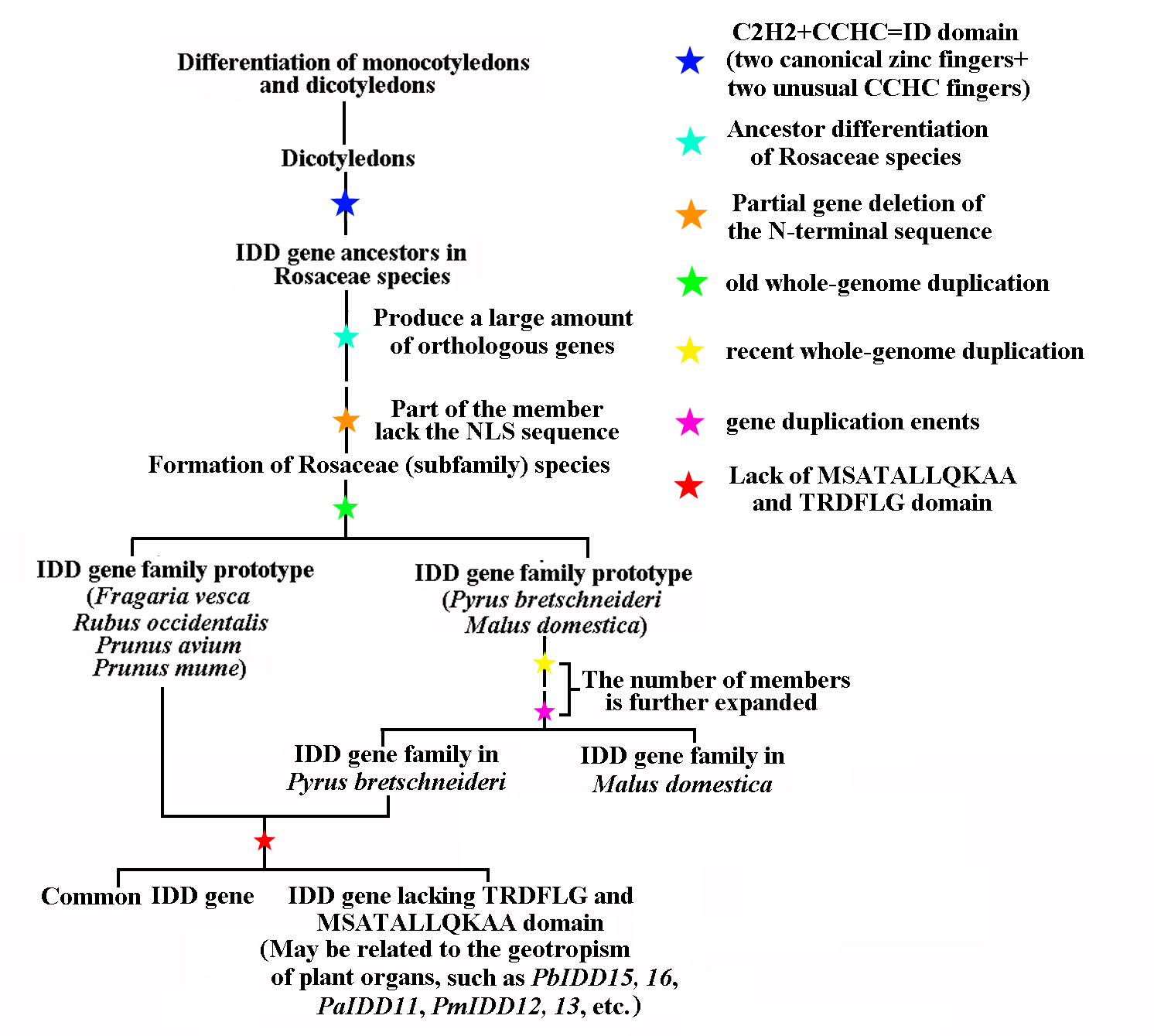

Supplement: Supplemental Information 10 [file peerj-07-6628-s010.png]

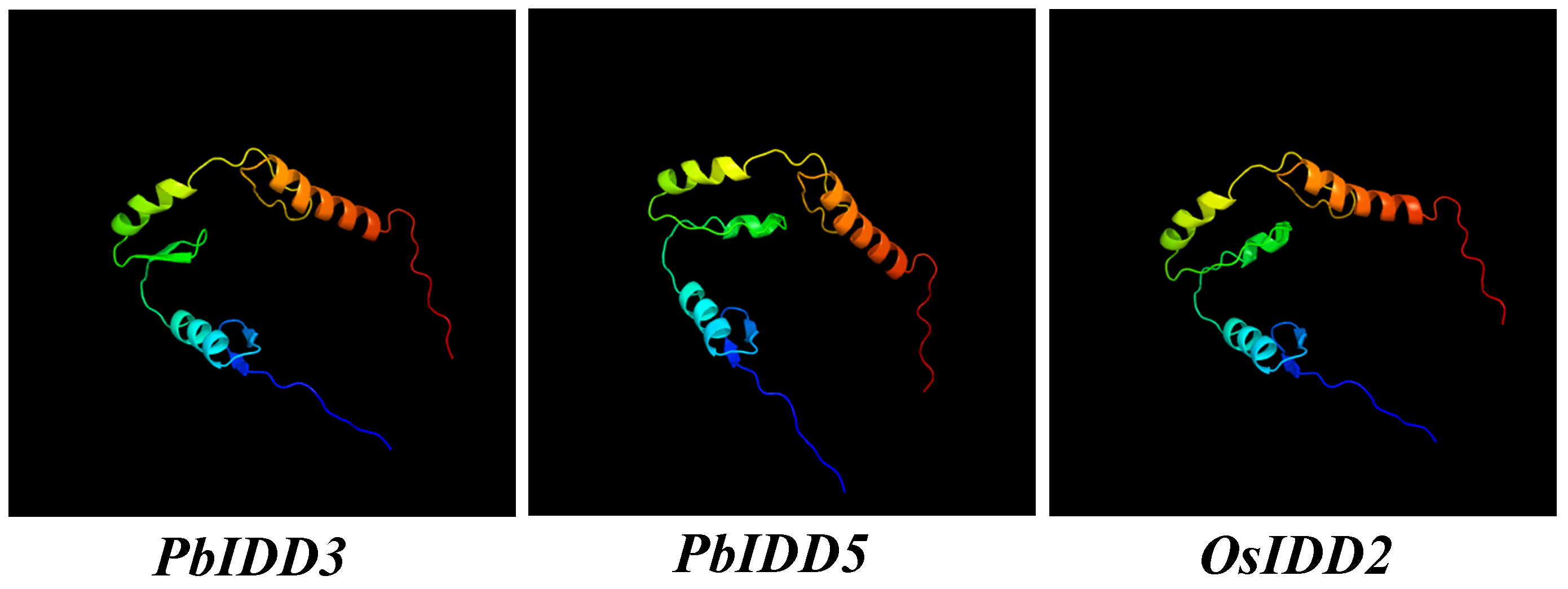

Supplement: Supplemental Information 11 — OsIDD2 have been proven to be responsible for SCW formation and lignin biosynthesis. [file peerj-07-6628-s011.png]
