# Supplementary material for: Comparative genomic analysis of the IDD genes in five Rosaceae species and expression analysis in Chinese white pear (Pyrus bretschneideri)
Source: PeerJ. 2019 Mar 26;7:e6628. doi: 10.7717/peerj.6628 (PMC6440465; doi:10.7717/peerj.6628)
Supplement: Supplemental Information 13 — The IDD genes of Fragaria vesca, Prunus mume, Rubus occidentalis and Prunus avium identified in this study are listed. [file peerj-07-6628-s013.docx]

**Supplemental Table S2. The IDD genes of strawberry, plum, raspberry and cherry identified in this study are listed.**

| **Gene name** | **Gene ID** | **AA** | **KD** | **pI** | **GRAVY** | **Preditced subcellular localization** |
| --- | --- | --- | --- | --- | --- | --- |
| *FvIDD1* | FvH4_1g13710.1 | 506 | 53.3 | 8.96 | -0.529 | nucl |
| *FvIDD2* | FvH4_2g40030.1 | 510 | 53.7 | 8.81 | -0.459 | nucl |
| *FvIDD3* | FvH4_6g33550.1 | 540 | 57.8 | 8.87 | -0.732 | nucl |
| *FvIDD4* | FvH4_6g15680.1 | 464 | 50.5 | 9.45 | -0.629 | nucl |
| *FvIDD5* | FvH4_6g06270.1 | 529 | 57.8 | 9.10 | -0.796 | nucl |
| *FvIDD6* | FvH4_4g36050.1 | 605 | 64.7 | 9.30 | -0.705 | nucl |
| *FvIDD7* | FvH4_2g31720.1 | 536 | 58.5 | 8.78 | -0.800 | nucl |
| *FvIDD8* | FvH4_3g32700.1 | 502 | 54.9 | 9.11 | -0.617 | nucl |
| *FvIDD9* | FvH4_2g22950.1 | 446 | 49.2 | 8.99 | -0.619 | nucl |
| *FvIDD10* | FvH4_c6g00070.1 | 493 | 53.8 | 8.99 | -0.695 | nucl |
| *FvIDD11* | FvH4_4g36040.1 | 492 | 52.9 | 9.05 | -0.753 | nucl |
| *FvIDD12* | FvH4_6g26760.1 | 425 | 46.9 | 9.19 | -0.731 | nucl |
| *FvIDD13* | FvH4_5g17840.1 | 496 | 55.4 | 8.76 | -0.837 | nucl |
| *FvIDD14* | FvH4_4g35830.1 | 471 | 52.2 | 8.55 | -0.871 | nucl |
| *PmIDD1* | pm__Pm026593 | 516 | 54.2 | 8.70 | -0.493 | nucl |
| *PmIDD2* | pm__Pm005795 | 509 | 53.1 | 8.78 | -0.414 | nucl |
| *PmIDD3* | pm__Pm013340 | 590 | 63.9 | 9.05 | -0.753 | nucl |
| *PmIDD4* | pm__Pm005951 | 606 | 65.2 | 9.24 | -0.742 | nucl |
| *PmIDD5* | pm__Pm030100 | 542 | 58.6 | 9.27 | -0.700 | nucl |
| *PmIDD6* | pm__Pm005517 | 525 | 57.7 | 8.48 | -0.780 | nucl |
| *PmIDD7* | pm__Pm001002 | 496 | 54.9 | 9.00 | -0.715 | nucl |
| *PmIDD8* | pm__Pm003292 | 728 | 79.6 | 9.28 | -0.545 | nucl |
| *PmIDD9* | pm__Pm020252 | 461 | 50.8 | 8.98 | -0.659 | nucl |
| *PmIDD10* | pm__Pm005950 | 631 | 67.8 | 8.82 | -0.752 | nucl |
| *PmIDD11* | pm__Pm014215 | 410 | 45.2 | 9.12 | -0.689 | nucl |
| *PmIDD12* | pm__Pm025274 | 505 | 56.8 | 8.80 | -0.871 | nucl |
| *PmIDD13* | pm__Pm005926 | 465 | 51.9 | 8.62 | -0.843 | nucl |
| *RoIDD1* | Bras_G01001 | 511 | 53.7 | 8.89 | -0.516 | nucl |
| *RoIDD2* | Bras_G02393 | 518 | 54.1 | 9.06 | -0.408 | nucl |
| *RoIDD3* | Bras_G18834 | 468 | 50.9 | 9.27 | -0.584 | nucl |
| *RoIDD4* | Bras_G09104 | 506 | 54.5 | 9.05 | -0.646 | nucl |
| *RoIDD5* | Bras_G15270 | 516 | 56.5 | 8.82 | -0.730 | nucl |
| *RoIDD6* | Bras_G04489 | 516 | 56.2 | 9.04 | -0.772 | nucl |
| *RoIDD7* | Bras_G04031 | 456 | 50.0 | 8.85 | -0.577 | nucl |
| *RoIDD8* | Bras_G07059 | 546 | 59.4 | 8.42 | -0.707 | nucl |
| *RoIDD9* | Bras_G25466 | 456 | 50.0 | 9.08 | -0.613 | nucl |
| *RoIDD10* | Bras_G00453 | 593 | 63.4 | 8.93 | -0.718 | nucl |
| *RoIDD11* | Bras_G00454 | 584 | 62.6 | 9.36 | -0.744 | nucl |
| *RoIDD12* | Bras_G07491 | 475 | 52.5 | 9.01 | -0.664 | nucl |
| *RoIDD13* | Bras_G03660 | 493 | 55.2 | 9.03 | -0.826 | nucl |
| *RoIDD14* | Bras_G00429 | 468 | 51.6 | 8.54 | -0.787 | nucl |
| *PaIDD1* | Pav_sc0001175.1 | 449 | 47.3 | 9.08 | -0.573 | nucl |
| *PaIDD2* | Pav_sc0002375.1 | 547 | 56.9 | 8.51 | -0.411 | nucl |
| *PaIDD3* | Pav_sc0000893.1 | 606 | 65.2 | 9.29 | -0.745 | nucl |
| *PaIDD4* | Pav_sc0000689.1 | 592 | 64.0 | 8.98 | -0.785 | nucl |
| *PaIDD5* | Pav_sc0000046.1 | 472 | 51.5 | 8.99 | -0.662 | nucl |
| *PaIDD6* | Pav_sc0000094.1 | 500 | 55.1 | 9.00 | -0.680 | nucl |
| *PaIDD7* | Pav_sc0006080.1 | 518 | 56.7 | 9.10 | -0.797 | nucl |
| *PaIDD8* | Pav_sc0000848.1 | 579 | 63.0 | 7.76 | -0.771 | nucl |
| *PaIDD9* | Pav_sc0000893.1 | 620 | 66.4 | 8.91 | -0.727 | nucl |
| *PaIDD10* | Pav_sc0000203.1 | 382 | 42.0 | 9.04 | -0.747 | nucl |
| *PaIDD11* | Pav_sc0000103.1 | 508 | 56.8 | 8.86 | -0.859 | nucl |
