# Supplementary material for: Comparative genomic analysis of the IDD genes in five Rosaceae species and expression analysis in Chinese white pear (Pyrus bretschneideri)
Source: PeerJ. 2019 Mar 26;7:e6628. doi: 10.7717/peerj.6628 (PMC6440465; doi:10.7717/peerj.6628)
Supplement: Supplemental Information 14 [file peerj-07-6628-s014.docx]

**Supplementary Table S3. Detailed information of the 20 motifs in the 68 IDD proteins.**

| Motif | Width | Sequences | Domain |
| --- | --- | --- | --- |
| 1 | 30 | WKCDKCSKKYAVQSDWKAHSKTCGTREYRC | ZF3 |
| 2 | 30 | RFVCEICNKGFQRDQNLQLHRRGHNLPWKL | ZF1 |
| 3 | 30 | VYVCPEPSCVHHDPSRALGDLTGIKKHFCR | ZF2 |
| 4 | 21 | DCGTLFSRRDSFITHRAFCDA | ZF4 |
| 5 | 30 | PAKKKRNLPGNPDPDAEVIALSPKTLMATN | * |
| 6 | 30 | QNNPPPSAHMSATALLQKAAQMGATTSSNS | C-terminal conserved motifs |
| 7 | 21 | GGGGDGLTRDFLGLGGGVGSD | C-terminal conserved motifs |
| 8 | 11 | KQRTSKEVKKK | * |
| 9 | 6 | KHGEKK | * |
| 10 | 15 | LAEESARLASALNAY | * |
| 11 | 21 | VDENMSNLTSASGEAASSSSG | * |
| 12 | 29 | AEEARRQAEHQIELAEKEFANAKRIRQQA | * |
| 13 | 15 | LSDLMNSLAGGGSSA | * |
| 14 | 27 | LKEQATKKVSSAILQITCHACKQHFHQ | * |
| 15 | 21 | SNSSSSSSLFASSFASSSTSS | * |
| 16 | 30 | QESLQWNQRZAEPDSASVAAGLGLGLPCDG | * |
| 17 | 21 | ACLSRTASSPSPSSDDNFSSR | * |
| 18 | 30 | DSTTDLAPSSSVEPISLSLSTSLYSSIFGS | * |
| 19 | 15 | LLRGFGLSSSSSASS | * |
| 20 | 21 | REENHSTQLQLSIGSCDFGGD | * |
