# Supplementary material for: Comparative genomic analysis of the IDD genes in five Rosaceae species and expression analysis in Chinese white pear (Pyrus bretschneideri)
Source: PeerJ. 2019 Mar 26;7:e6628. doi: 10.7717/peerj.6628 (PMC6440465; doi:10.7717/peerj.6628)
Supplement: Supplemental Information 15 [file peerj-07-6628-s015.docx]

**Supplementary Table S4. Ka/Ks analysis of the duplicated IDD paralogues from Chinese white pear.**

| **Duplicated Pairs** | **Ka** | **Ks** | **Ka/Ks** | **Purifying Selection** | **Duplicated type** |
| --- | --- | --- | --- | --- | --- |
| ***PbIDD3-PbIDD5*** | 0.0413 | 0.2017 | 0.2048 | Yes | Segmental |
| ***PbIDD4-PbIDD11*** | 0.0450 | 0.1939 | 0.2321 | Yes | Segmental |
| ***PbIDD9-PbIDD10*** | 0.0399 | 0.1957 | 0.2039 | Yes | Segmental |
| ***PbIDD13-PbIDD14*** | 0.0540 | 0.1981 | 0.2726 | Yes | Segmental |
