# Supplementary material for: Comparative genomic analysis of the IDD genes in five Rosaceae species and expression analysis in Chinese white pear (Pyrus bretschneideri)
Source: PeerJ. 2019 Mar 26;7:e6628. doi: 10.7717/peerj.6628 (PMC6440465; doi:10.7717/peerj.6628)
Supplement: Supplemental Information 16 — Synteny data in Pyrus bretschneideri, Fragaria vesca, Prunus mume, Rubus occidentalis, Prunus avium, Malus domestica. [file peerj-07-6628-s016.docx]

**Supplemental Table S5. Synteny data in pear, strawberry, plum, raspberry, cherry, apple**

| Numble | Segment1 | Segment2 |
| --- | --- | --- |
| Segdup1 | *PbIDD1* | *FvIDD1* |
| Segdup2 | *PbIDD1* | *MdIDD1* |
| Segdup3 | *PbIDD2* | *MdIDD8* |
| Segdup4 | *PbIDD4* | *MdIDD5* |
| Segdup5 | *PbIDD6* | *MdIDD18* |
| Segdup6 | *PbIDD7* | *MdIDD19* |
| Segdup7 | *PbIDD8* | *MdIDD6* |
| Segdup8 | *PbIDD9* | *MdIDD12* |
| Segdup9 | *PbIDD10* | *MdIDD3* |
| Segdup10 | *PbIDD11* | *MdIDD14* |
| Segdup11 | *PbIDD12* | *MdIDD20* |
| Segdup12 | *PbIDD13* | *MdIDD10* |
| Segdup13 | *PbIDD14* | *MdIDD2* |
| Segdup14 | *PbIDD1* | *RoIDD1* |
| Segdup15 | *PbIDD9* | *RoIDD7* |
| Segdup16 | *PbIDD10* | *RoIDD7* |
| Segdup17 | *PbIDD1* | *PmIDD1* |
| Segdup18 | *PbIDD2* | *PmIDD2* |
| Segdup19 | *PbIDD3* | *PmIDD3* |
| Segdup20 | *PbIDD6* | *PmIDD6* |
| Segdup21 | *PbIDD9* | *PmIDD9* |
| Segdup22 | *PbIDD10* | *PmIDD9* |
| Segdup23 | *PbIDD12* | *PmIDD10* |
| Segdup24 | *PbIDD15* | *PmIDD12* |
| Segdup25 | *PbIDD1* | *PaIDD1* |
| Segdup26 | *PbIDD2* | *PaIDD2* |
| Segdup27 | *PbIDD3* | *PaIDD4* |
| Segdup28 | *PbIDD7* | *PaIDD3* |
| Segdup29 | *PbIDD8* | *PaIDD7* |
| Segdup30 | *PbIDD15* | *PaIDD11* |

The Identifier of the gene corresponding to the gene name was shown in supplemental table S1.
