# Supplementary material for: Comparative genomic analysis of the IDD genes in five Rosaceae species and expression analysis in Chinese white pear (Pyrus bretschneideri)
Source: PeerJ. 2019 Mar 26;7:e6628. doi: 10.7717/peerj.6628 (PMC6440465; doi:10.7717/peerj.6628)
Supplement: Supplemental Information 18 [file peerj-07-6628-s018.docx]

**Supplementary Table S7. Complete information on *Cis*-acting elements of the 16 *PbIDD* genes.**

| Site Name | Organism | Matrix score | Sequence | Function |
| --- | --- | --- | --- | --- |
| ABRE | *Triticum aestivum* | 10 | GACACGTGGC | cis-acting element involved in the abscisic acid responsiveness |
| CGTCA-motif | *Hordeum vulgare* | 5 | CGTCA | cis-acting regulatory element involved in the MeJA-responsiveness |
| TGACG-motif | *Hordeum vulgare* | 5 | TGACG | cis-acting regulatory element involved in the MeJA-responsiveness |
| P-box | *Oryza sativa* | 7 | CCTTTTG | gibberellin-responsive element |
| GARE-motif | *Brassica oleracea* | 7 | TCTGTTG | gibberellin-responsive element |
| TGA-element | *Brassica oleracea* | 6 | AACGAC | auxin-responsive element |
| TCA-element | *Brassica oleracea* | 10 | TCAGAAGAGG | cis-acting element involved in salicylic acid responsiveness |
| CAT-box | *Arabidopsis thaliana* | 6 | GCCACT | cis-acting regulatory element related to meristem expression |
| CCGTCC-box | *Arabidopsis thaliana* | 6 | CCGTCC | cis-acting regulatory element related to meristem specific activation |
| G-box | *Zea mays* | 6 | CACGTC | cis-acting regulatory element involved in light responsiveness |
| HSE | *Brassica oleracea* | 10 | AGAAAATTCG | cis-acting element involved in heat stress responsiveness |
| MBS | *Arabidopsis thaliana* | 6 | CAACTG | MYB binding site |
| TC-rich repeats | *Nicotiana tabacum* | 10 | ATTTTCTCCA | cis-acting element involved in defense and stress responsiveness |
