# Supplementary material for: Comparative genomic analysis of the IDD genes in five Rosaceae species and expression analysis in Chinese white pear (Pyrus bretschneideri)
Source: PeerJ. 2019 Mar 26;7:e6628. doi: 10.7717/peerj.6628 (PMC6440465; doi:10.7717/peerj.6628)
Supplement: Supplemental Information 19 [file peerj-07-6628-s019.docx]

**Supplementary Table S8. Primers used in qRT-PCR.**

| Gene name | Primer sequences 5’ | Primer sequences 3’ |
| --- | --- | --- |
| *PbIDD1* | CAAGTTGTAGAAGAAGTG | ATGTTGTTGAGGAAGTAA |
| *PbIDD2* | TTAACTACCACGGCAATC | ATACACTGGCGAATACAC |
| *PbIDD3* | AGTGACATTCTGAACATTG | GAAGCAGTATTAGCAACAG |
| *PbIDD4* | AGGAGGAGGAGAACATATTG | TAGTGGTTGCGAGTGAAT |
| *PbIDD5* | GCTGCTAGTGCCTTATCT | CTTGTGGTGGTGTGGTAA |
| *PbIDD6* | AGTATAGATGTGATTGTGGAA | TGTATTGTTCTCTTGTGCTA |
| *PbIDD7* | TTCCAAGCAAGGCATTAC | AGAGATTCGCAGCACTAT |
| *PbIDD8* | TACAGGTGGAGAAGAAGTT | GTTGGAAGGAAGGAGTTG |
| *PbIDD9* | TCAATGGCGATGGCACTG | ACGGTAGCATGTCCTTAACG |
| *PbIDD10* | TTCTCCTGTCCACCAATA | GCAATCACTTCAGCATCT |
| *PbIDD11* | TAGGCTCGCTACTTCATC | ACTACTGCTTGACTATTGTTC |
| *PbIDD12* | ATCCAACCAACAACAATACCA | CGGAGTGATGCTGATGTG |
| *PbIDD13* | TCTATGTCTTCCTCGTCAA | CTGGCATCATCAAGTGTT |
| *PbIDD14* | GAGAGTATAGATGTGACT | CTTGGATGAATAGTTGAA |
| *PbIDD15* | AGAGAATAAGGCAACAAG | TTGGAGAATGGTAGAGTT |
| *PbIDD16* | CACGCAACTAATCTGAAG | CGGACGATGAATTATACTTG |
| *Tubulin* | AGAACAAGAACTCGTCCTAC | GAACTGCTCGCTCACTCTCC |
