# Supplementary material for: Comparative genomic analysis of the IDD genes in five Rosaceae species and expression analysis in Chinese white pear (Pyrus bretschneideri)
Source: PeerJ. 2019 Mar 26;7:e6628. doi: 10.7717/peerj.6628 (PMC6440465; doi:10.7717/peerj.6628)
Supplement: Supplemental Information 20 [file peerj-07-6628-s020.docx]

**Supplemental Table S9.** GO annotations analysis of all 68 IDD genes.

| Gene name | InterPro GO IDs | InterPro GO Names |
| --- | --- | --- |
| *PbIDD1* | GO: 0003676 | Nucleic acid binding |
| *PbIDD2* | GO: 0003676 | Nucleic acid binding |
| *PbIDD3* | GO: 0003676 | Nucleic acid binding |
| *PbIDD4* | GO: 0003676, 0003700 | Nucleic acid binding; DNA-binding transcription factor activity |
| *PbIDD5* | GO: 0003676 | Nucleic acid binding |
| *PbIDD6* | GO: 0003676 | Nucleic acid binding |
| *PbIDD7* | GO: 0003676 | Nucleic acid binding |
| *PbIDD8* | GO: 0003676 | Nucleic acid binding |
| *PbIDD9* | GO: 0003676 | Nucleic acid binding |
| *PbIDD10* | GO: 0003676 | Nucleic acid binding |
| *PbIDD11* | GO: 0003676, 0003700 | Nucleic acid binding; DNA-binding transcription factor activity |
| *PbIDD12* | GO: 0003676 | Nucleic acid binding |
| *PbIDD13* | GO: 0003676, 0003700 | Nucleic acid binding; DNA-binding transcription factor activity |
| *PbIDD14* | GO: 0003676, 0003700 | Nucleic acid binding; DNA-binding transcription factor activity |
| *PbIDD15* | GO: 0003676 | Nucleic acid binding |
| *PbIDD16* | GO: 0003676, 0003700 | Nucleic acid binding; DNA-binding transcription factor activity |
| *FvIDD1* | GO: 0003676 | Nucleic acid binding |
| *FvIDD2* | GO: 0003676 | Nucleic acid binding |
| *FvIDD3* | GO: 0003676 | Nucleic acid binding |
| *FvIDD4* | GO: 0003676, 0003700 | Nucleic acid binding; DNA-binding transcription factor activity |
| *FvIDD5* | GO: 0003676 | Nucleic acid binding |
| *FvIDD6* | GO: 0003676 | Nucleic acid binding |
| *FvIDD7* | GO: 0003676 | Nucleic acid binding |
| *FvIDD8* | GO: 0003676, 0003700 | Nucleic acid binding; DNA-binding transcription factor activity |
| *FvIDD9* | GO: 0003676 | Nucleic acid binding |
| *FvIDD10* | GO: 0003676 | Nucleic acid binding |
| *FvIDD11* | GO: 0003676 | Nucleic acid binding |
| *FvIDD12* | GO: 0003676 | Nucleic acid binding |
| *FvIDD13* | GO: 0003676 | Nucleic acid binding |
| *FvIDD14* | GO: 0003676, 0003700 | Nucleic acid binding; DNA-binding transcription factor activity |
| *PmIDD1* | GO: 0003676 | Nucleic acid binding |
| *PmIDD2* | GO: 0003676 | Nucleic acid binding |
| *PmIDD3* | GO: 0003676 | Nucleic acid binding |
| *PmIDD4* | GO: 0003676 | Nucleic acid binding |
| *PmIDD5* | GO: 0003676 | Nucleic acid binding |
| *PmIDD6* | GO: 0003676 | Nucleic acid binding |
| *PmIDD7* | GO: 0003676, 0003700 | Nucleic acid binding; DNA-binding transcription factor activity |
| *PmIDD8* | GO: 0003676 | Nucleic acid binding |
| *PmIDD9* | GO: 0003676 | Nucleic acid binding |
| *PmIDD10* | GO: 0003676 | Nucleic acid binding |
| *PmIDD11* | GO: 0003676 | Nucleic acid binding |
| *PmIDD12* | GO: 0003676 | Nucleic acid binding |
| *PmIDD13* | GO: 0003676 | Nucleic acid binding |
| *RoIDD1* | GO: 0003676 | Nucleic acid binding |
| *RoIDD2* | GO: 0003676 | Nucleic acid binding |
| *RoIDD3* | GO: 0003676, 0003700 | Nucleic acid binding; DNA-binding transcription factor activity |
| *RoIDD4* | GO: 0003676 | Nucleic acid binding |
| *RoIDD5* | GO: 0003676 | Nucleic acid binding |
| *RoIDD6* | GO: 0003676 | Nucleic acid binding |
| *RoIDD7* | GO: 0003676 | Nucleic acid binding |
| *RoIDD8* | GO: 0003676 | Nucleic acid binding |
| *RoIDD9* | GO: 0003676 | Nucleic acid binding |
| *RoIDD10* | GO: 0003676 | Nucleic acid binding |
| *RoIDD11* | GO: 0003676 | Nucleic acid binding |
| *RoIDD12* | GO: 0003676, 0003700 | Nucleic acid binding; DNA-binding transcription factor activity |
| *RoIDD13* | GO: 0003676 | Nucleic acid binding |
| *RoIDD14* | GO: 0003676 | Nucleic acid binding |
| *PaIDD1* | GO: 0003676 | Nucleic acid binding |
| *PaIDD2* | GO: 0003676 | Nucleic acid binding |
| *PaIDD3* | GO: 0003676 | Nucleic acid binding |
| *PaIDD4* | GO: 0003676 | Nucleic acid binding |
| *PaIDD5* | GO: 0003676, 0003700 | Nucleic acid binding; DNA-binding transcription factor activity |
| *PaIDD6* | GO: 0003676 | Nucleic acid binding |
| *PaIDD7* | GO: 0003676 | Nucleic acid binding |
| *PaIDD8* | GO: 0003676 | Nucleic acid binding |
| *PaIDD9* | GO: 0003676 | Nucleic acid binding |
| *PaIDD10* | GO: 0003676 | Nucleic acid binding |
| *PaIDD11* | GO: 0003676 | Nucleic acid binding |
